# A reusable learning object for assessment cardiovascular and respiratory responses*

**DOI:** 10.17533/udea.iee.v40n2e10

**Published:** 2022-09-19

**Authors:** Leidy Johanna Rueda Díaz, David Alejandro Mercado Miranda, Clara Inés Padilla García

**Affiliations:** 1 Ph.D. Associate Professor, School of Nursing. Universidad Industrial de Santander, Bucaramanga, Colombia Email: ljruedad@uis.edu.co Universidad Industrial de Santander School of Nursing Universidad Industrial de Santander Bucaramanga Colombia ljruedad@uis.edu.co; 2 Ph.D. Full Professor, School of Physics. Universidad Industrial de Santander, Bucaramanga, Colombia Email: dalemir@uis.edu.co Universidad Industrial de Santander School of Physics Universidad Industrial de Santander Bucaramanga Colombia dalemir@uis.edu.co; 3 Master's degree. Assistant Professor, School of Nursing. Universidad Industrial de Santander, Bucaramanga, Colombia Email: cipadiga@uis.edu.co Universidad Industrial de Santander School of Nursing Universidad Industrial de Santander Bucaramanga Colombia cipadiga@uis.edu.co

**Keywords:** Nursing, Distance Education, Educational Technology, Enfermería, Educación a Distancia, Tecnología Educativa., Enfermagem, Educação a Distância, Tecnologia Educacional

## Abstract

**Objective.:**

Produce and determine the validity of a reusable learning object for assessment cardiovascular and respiratory responses from the taxonomy of the North American Association of Nursing Diagnosis Domain 4. Activity/Rest, Class 4. Cardiovascular/Pulmonary Responses.

**Methods.:**

A descriptive methodological study was developed that included three phases (1) construction of the reusable learning object incorporating Gagné's nine instructional events, (2) content validation was carried out with 24 nurses who served as experts, and (3) and Usability was evaluated by 22 nursing students from a Public University in Colombia.

**Results.:**

The reusable learning object was organized into three modules: introduction, assessment of cardiovascular responses, and assessment of pulmonary responses. The learning object obtained a content validation index of 0.86; the usability indicators had proportions of agreement greater than 85%.

**Conclusion.:**

The reusable learning object is valid and can be used for teaching the assessment of cardiovascular and respiratory responses in nursing students.

## Resumen

**Objetivo.:**

Producir y determinar la validez de un objeto de aprendizaje reutilizable para la evaluación de las respuestas cardiovasculares y respiratorias de la taxonomía de la Asociación Norteamericana de Diagnóstico de Enfermería Dominio 4. Actividad/Descanso, Clase 4. Respuestas Cardiovasculares/Pulmonares.

**Métodos.:**

Fue desarrollado un estudio metodológico descriptivo que incluyó tres fases (1) construcción del objeto de aprendizaje reutilizable incorporando los nueve eventos de instrucción de Gagné, (2) validación de contenido con 24 enfermeras que fungieron como expertas, y (3) evaluación de la usabilidad por 22 estudiantes de enfermería de una Universidad Pública en Colombia.

**Resultados.:**

El objeto de aprendizaje reutilizable se organizó en tres módulos: introducción, valoración de las respuestas cardiovasculares y valoración de las respuestas pulmonares. El objeto de aprendizaje obtuvo un índice de validación de contenido de 0.86; además, los indicadores de usabilidad tuvieron proporciones de acuerdo superiores al 85%**.**

**Conclusión.:**

El objeto de aprendizaje reutilizable desarrollado es válido y puede ser empleado para la enseñanza de la valoración de las respuestas cardiovasculares y respiratorias en estudiantes de enfermería.

## Resumo

**Objetivo.:**

Produzir e determinar a validade de um objeto de aprendizagem reutilizável para avaliar respostas cardiovasculares e respiratórias a partir da taxonomia da Associação Norte-Americana de Diagnósticos de Enfermagem Domínio 4. Atividade/Repouso, Classe 4. Respostas Cardiovasculares/Pulmonares.

**Métodos.:**

Foi desenvolvido um estudo metodológico descritivo que incluiu três fases (1) construção do objeto de aprendizagem reutilizável incorporando os nove eventos instrucionais de Gagné, (2) validação de conteúdo realizada com 24 enfermeiros que atuaram como especialistas e (3) avaliacão da usabilidade por 22 estudantes de enfermagem de uma universidade pública da Colômbia.

**Resultados.:**

O objeto de aprendizagem reutilizável foi organizado em três módulos: introdução, avaliação das respostas cardiovasculares e avaliação das respostas pulmonares. O objeto de aprendizagem obteve índice de validação de conteúdo de 0,86; os indicadores de usabilidade tiveram proporções de concordância superiores a 85%.

**Conclusão.:**

O objeto de aprendizagem reutilizável é válido e pode ser utilizado para o ensino da avaliação das respostas cardiovasculares e respiratórias em estudantes de enfermagem**.**

## Introduction

Assessment is the first step in the application of the nursing process and involves critical thinking skills for data collection.^1^ The ability to assess the state of health is a competence of great importance for nursing students and nurses alike. The obtained information allows for the correct identification of care needs for healthy or sick people. In the Nursing program at the Industrial University of Santander (UIS), Colombia, the health status assessment is carried out within the framework of the taxonomy II of the North American Nursing Diagnosis Association (NANDA-I);^2^ for the implementation of this framework, an assessment form was created in 2008, which included the thirteen domains or categories of nursing practice. In the NANDA Domain 4. Activity/Rest, Cardiovascular and Pulmonary Responses are addressed as class 4.

In our teaching experience, we observed that nursing students have difficulties in learning and assessing these responses. Although scarce, some studies available in the scientific literature show that teachers have problems in the teaching pulmonary and cardiovascular assessment-mainly in what has to do with auscultation;^3^ as well as, nursing students have difficulties in cardiac auscultation and the identification of the sounds.^4^ To address these difficulties, it is necessary to generate conditions so that the nursing student can develop the ability to perform accurate assessments. Subsequently, as professionals, this will result in the improvement of health outcomes and quality of patient care. It is important to notice that nurses with higher levels of assessment skills have a greater capacity to monitor the changes in the health of the people they care for.^5^

The results from a cohort study that aimed to investigate the risk of mortality associated with nurses’ assessments of patients by physiological systems, including respiratory and cardiovascular systems, showed that patients whose nursing assessments at admission did not meet the minimum standards had significantly higher hospital mortality than those patients who had assessments that met the minimum.^6^ The authors concluded that the results show evidence of the clinical validity of nursing assessments as well as the fact that they can help with medical care and possibly reduce the mortality of hospitalized patients;^6^ also, we recognized that the deterioration of the physiological state is often unappreciated or acted upon in a timely manner.^7^,^8^ Evidence suggests that health personnel may lack the knowledge and skills necessary to perform a respiratory and cardiovascular assessment; this ultimately has a detrimental effect on the potential to minimize adverse patient events;^9^,^10^ therefore, encouraging nursing students to develop skills for assessing health status is a matter of interest to the discipline.

In recent years, educational technologies to mediate nursing teaching have transformed the way students learn since they went from passive receivers of information to protagonists of their learning. With its implementation, a greater dynamism is generated in the traditional classroom method, and the students' interest is favored.^11^ However, incorporating these technologies requires a change in the educational paradigm,^12^ teacher training, and investment in technological infrastructure.

Educational technologies such as reusable learning objects (RLOs) have been introduced to assist in the learning process in nursing education.^13^ The RLO is an interactive, multimedia web-based resource focused on a single learning objective that can be used in multiple contexts; it focuses on a specific topic and is highly visual with an auditory component and high-quality graphics.^14^,^15^ This resource encourages active and meaningful learning in students and changes the relationship between the student and the content objective for study.^13^ The use of RLOs has proven to be an innovative, constructive, and interactive educational experience for nursing students, similar to real situations faced in a healthcare setting, appealing for their significant learning.^16^

Some studies have produced and validated RLO for teaching medication administration,^17^ vital signs,^17^ the systematization of nursing care,^17^ semiotics and neonatal semiology,^17^,^18^ anatomy,^17^ pain assessment^15^ and nursing care of intestinal elimination stoma.^19^ Of these, two studies^15^,^18^ report positive results for student learning when exposed to an educational intervention based on the RLO. We emphasize that, to date, there are no published studies that report the construction and evaluation of any virtual object for teaching the assessment of the NANDA Domain 4. Activity/Rest, class 4 Cardiovascular and Pulmonary Response.

Because of the need for new resources in teaching nursing students to quickly and easily master the content related to pulmonary and cardiovascular responses assessment, this study aimed to produce and determine the validity of an RLO for assessment of the NANDA Domain 4. Activity/Rest, class 4 Cardiovascular and Pulmonary Responses. The following research question was formulated: What is the validity of an RLO for assessing the NANDA Domain 4. Activity/Rest, class 4 Cardiovascular and Pulmonary Responses?

## Methods

A descriptive methodological study was conducted between December 2017 and December 2018, with sequential phases that included: (I) construction of the reusable learning object, (II) content validation by nurses, and (III) evaluation of usability by students of the nursing program of the UIS; It is a non-profit public higher education institution located in the urban setting of the medium-sized city of Bucaramanga, Colombia.

In phase I, we adopted the first three steps of the methodology proposed by Mendoza-Galvis^20^ to create virtual learning environments; these were analysis, design, and development. In the analysis step, we characterized the target audience and defined the learning objectives, modules, and content of the RLO by examining relevant literature in databases, virtual libraries, and books about assessing health status. In the design step, we produced the theoretical content in the Word processor and developed support resources for approaching the subject in this stage, such as drawings, videos, and photographs. Also, we incorporated Gagné's nine events of instruction; the way it was done will be presented in the results section. This instructional design model was applied because it provides a formal template that gives structure to the lesson to achieve learning objectives.^21^ Finally, in the development step, a graphic designer built the RLO and integrated the content and resources produced in the previous step using a standard programming language (HTMl5) with Java Scripts and CSS3. The illustration was created with Adobe Illustrator.

In phase II, 22 nurses who served as experts carried out the content validation; the number of experts was calculated by Equation (1) which was proposed by Lopes and collaborators^22^ in the context of content validation of nursing diagnosis to get the proportion of experts who agree upon the inclusion of a given component (for example, clinical indicator) for a specific diagnosis.

$n=Z_{\alpha \;}^{2}\frac{P\;(1-P)}{e^{2}}$

In Equation (1), $Z_{\alpha \;}^{2}$ refers to the confidence level adopted, *P* corresponds to the expected proportion of the nurses reporting the suitability of each component evaluated (objectives, content, relevance, and environment) for the RLO, and *e* ^*2*^ represents the acceptable proportional difference about what would be expected.^22^ For this study, we adopted a confidence level 95%, a coefficient Z of 1.96 according to the standard normal distribution, and an expected expert proportion of 85% with a 15% margin of error, indicating that at least 70% of nurses who participated in the content validation would have to rate the component evaluated as suitable.^22^

The inclusion criteria were (I) to be a nursing professional, (II) to have at least two years of experience in teaching the assessment of health status, and (III) to have at least 2 years of clinical experience in cardiorespiratory care; the search for specialists was through social contact; there were no exclusion criteria.

In phase 3. the nursing students evaluated the usability RLO; the usability was defined as the capability of understanding, learning, using, and being attractive to the user, when used under specified conditions.^23^ The aspects contemplated in this evaluation were language, design, content, interaction, and stimulus; the sample size was calculated applying the same criteria used for the calculation of the nurses; therefore, the sample size was 22 nursing students. Inclusion criteria adopted for students were (I) to be enrolled in the Nursing program and (II) having taken and passed the subject nursing process II; there were no exclusion criteria. The students evaluated the usability RLO. Usability was defined as the capability of understanding, learning, using, and being attractive to the user, when used under specified conditions;^16^ the aspects contemplated in this evaluation were language, design, content, interaction, and stimulus.

According to the above, two instruments were used to collect the data; one was applied to nurses in phase 2, and the other was applied to nursing students in phase 3. Both were adapted from other published studies on validation.^24^^-^26 The instrument of nurses was divided into two parts: the first contained the characterization question, and the second contained the content validation questionnaire (33 questions). The instrument of nursing students was also divided into two parts: the first with the sociodemographic question, and the second, the usability questionnaire (21 questions). For both questionnaires, a Likert scale with five response options was used, being: (1) totally inadequate, (2) considerably inadequate, (3) somewhat adequate, (4) considerably adequate, and (5) totally adequate.

Besides, nurses and nursing students were invited to participate in the study in person and by email; they responded to the self-applied instruments after signing the informed consent. The instruments were filled after navigating freely (for approximately 3 hours) through the RLO in a computer classroom of the university. The data were processed in Microsoft Office Excel and analyzed using the Statistical Package for the Social Sciences (SPSS), version 25; the characterization variables of the nurses and nursing students were analyzed using descriptive statistics. The content validation process by nurses used two methods.

First, the Fehring method,^27^ which has been used to validate the content of diagnosis nursing of the NANDA-I and outcomes of The Nursing Outcomes Classification (NOC), allowed to verify how suitable were the components evaluated in regard to LO; for this, the experts ‘rating received a weight: (1) totally inadequate = 0, (2) considerably inadequate = 0.25, (3) somewhat adequate = 0.50, (4) considerably adequate = 0.75, (5) totally adequate = 1. Then, the weighted averages for each component evaluated were calculated. To calculate content validity index of the RLO (IVC) we added the weighted averages and divided them by the total number of components evaluated; components with weighted average equal to or higher than 0.78 were considered valid.^28^

Second, the proportion of experts who agreed with the relevance of the components evaluated was verified using an analysis of proportions by means of the binomial test; for this analysis, the responses were grouped dichotomously. The frequencies of responses concerning options (1), (2), and (3) of the Likert scale were classified as not relevant; (4) and (5) as relevant. The level of significance adopted was <0.05, so values above 0.05 indicated that the proportion that considered the appropriate item was statistically not less than 70%; in the case of usability, we also performed an analysis of proportions with the same criteria. After receiving the validation instruments completed by the nurses and students, the need to adjust the RLO was verified; this made it possible to obtain a final version of the RLO.

This study was developed in accordance with resolution 008430 of October 4/1993, which establishes the scientific, technical, and administrative standards for health research in Colombia. The *Comité de Ética en Investigación Científica* (CEINCI) of the UIS, Colombia, approved this study. All participants in this study signed the informed consent form.

## Results

The results obtained in this research work are shown below.

### Construction of the reusable learning object

We determined that the population of the RLO would be nursing students from a public university in Colombia. We also proposed, as learning objectives, that once the student interacted with the RLO, he/she would be able to a) integrate theoretical concepts of anatomy and physiology with the practice of assessing cardiovascular and pulmonary responses, b) correctly execute the techniques of assessment applied to Domain 4. Activity/Rest, cardiovascular and pulmonary responses.

**Figure 1:**
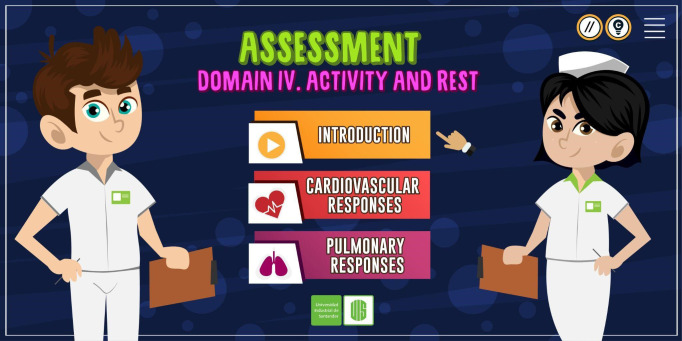
Starting screen.

**Figure 2:**
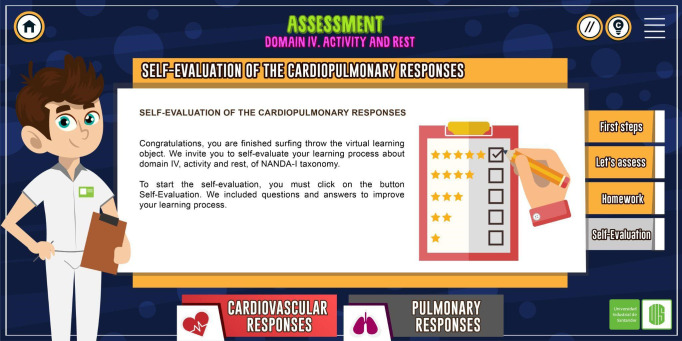
Section assessment.

The RLO was organized into three modules: introduction, assessment of cardiovascular responses, and assessment of pulmonary responses (Figure 1). The last two modules were subdivided, respectively, into three sections: a review of anatomy and physiology, assessment (interview, inspection, palpation, percussion, and auscultation), and tasks; the RLO offered interactive resources, including the possibility of listening to the chest with a stethoscope for learning to identify normal and abnormal heart and lung sounds, and a self-assessment questionnaire. Its exercises provided immediate feedback to the student as, at the end of the activities, correct answers can be verified (Figure 2). Gagné’s nine events of instruction were incorporated in the RLO as follows (Table 1).

**Table 1:** Operationalization of instruction events proposed by Robert Gagné

Events of instruction	Operationalization
Inform students of the objectives	The learning objectives were specified in the introduction.
Gain attention of the students	Images of the respiratory and cardiovascular system, and audios of normal *and abnormal* breath/heart *sounds* were recorded and implemented in the LO. In the texts drafted, the main elements that should be learned by the students were highlighted.
Stimulate recall of prior learning	A chart called "Key Points" was created to remind students of the relevant aspects addressed in the LO.
Present the content	An introduction was drafted. It explained the contents and resources of the LO as well as how to use it.
Provide learning guidance	Videos demonstrating the procedures that students must perform to assess pulmonary and cardiovascular responses were developed. The videos were supported by texts.
Elicit performance (practice)	Simulation exercises were proposed in the section “Task” for the student to practice at home.
Provide feedback	In the self-assessment section, when the questions are not answered correctly, messages are displayed explaining the correct option or answer.
Assess performance	A section of self-assessment exercises was created where ‘true or false’ and multiple-choice questions should be answered. At the end of answering the self-evaluations, a report of the qualification obtained is presented.
Enhance retention and transfer to the job	The development of tasks and evaluation by the students also stimulates retention.

### Content validation by nurses

26 Nurses were identified and invited to participate, 24 of whom agreed to participate. Then, the sample was composed of 24 nurses; the majority were female (75%), with a specialization in critical care nursing (46%) and/or a master's degree in nursing (54%). The time of teaching experience in assessing health status and clinical experience in cardiorespiratory care were, on average nine years (SD=5 years) and 13 years (SD=6 years), respectively. The results of content validation are shown in Table 2.

**Table 2:** Validation content by Nursing experts (n=24)

Evaluated aspects	WA*	% Agree	*p*-value**
Objectives			
Consistent with nursing practice	0.91	88	0.50
Objectives can be achieved	0.93	96	0.10
Content			
The content is consistent with the proposed objectives	0.85	88	0.50
The content is consistent with nursing practice	0.93	96	0.11
The content is presented in a logical sequence	0.90	92	0.28
The content addresses precisely the assessment	0.80	83	0.49
The information presented is correct and updated	0.89	88	0.51
The images illustrate correctly what was mentioned in the text	0.80	83	0.49
The videos are compatible with the reality	0.89	100	1.00
The audios are compatible with the reality	0.84	83	0.49
The content is adequate for students	0.88	92	0.28
The language used is understandable	0.89	83	0.49
The RLO has a sufficient number of topics, properly divided	0.86	83	0.49
The content has all the necessary resources to demonstrate the assessment	0.79	75	0.14
The content has all the necessary steps to carry out the assessment	0.84	83	0.49
Relevance			
The RLO is relevant for nursing practice	0.97	100	1.00
The homework is relevant so that the student can complement their knowledge	0.80	75	0.14
The images illustrate important aspects for the assessment	0.83	83	0.49
The videos illustrate important aspects for the assessment	0.87	91	0.31
The audios reproduce important sounds for assessment	0.82	83	0.49
The images are relevant for students can make the assessment	0.82	83	0.49
The videos are relevant for students can make the assessment	0.85	83	0.49
The audios are relevant for students can make the assessment	0.83	79	0.29
Environment			
The virtual object is adequate for content presentation	0.89	88	0.51
The images used are adequate for learning	0.80	79	0.29
The videos used are adequate for learning	0.86	83	0.49
The homework provides learning situations	0.81	71	0.06
The evaluation favors learning	0.81	75	0.14
**Content validity index**	0.86		

*Weighted averages

**Binomial test

The IVC was 0.86, and all the components of the RLO obtained a weighted average equal to or higher than 0.78, together with a minimum proportion of 85% of agreement on the relevance of each component evaluated (p>0.05); therefore, the RLO was considered valid in its content. In this phase, the experts recommended the replacement of the image about palpation of the femoral pulse, standardization of typing and repeating expression, and revision of the redaction of the self-assessment exercises. The authors accepted all these recommendations. 

### Evaluation of usability by nursing students

Twenty-two students participated in this phase, with a predominance of women (91%), with a mean age of 22 years (SD = 1.7); the majority of the students were studying in their seventh semester of Nursing (55%). The results of the usability evaluation by the students are shown in Table 3. All the evaluated components obtained a minimum proportion of 85% agreement among the nursing students on the relevance of each evaluated component (p>0.05), it is inferred that the RLO can be understood and engaging for the students who use it. As with the nurses, the nursing student suggested reviewing the redaction of the self-assessment exercises as well as the veracity of the answers to these exercises. The authors accepted the recommendations resulting from phase 3.

**Table 3:** Evaluation of the usability of the virtual object by Nursing students (n=22)

Aspects evaluated	n	% Agree	*p*-value*
Language			
The language is appropriate for the students	22	100	1.000
The language is easy to understand	22	100	1.000
Design			
The amount of information on each page is appropriate	17	77	0.226
The colors are appropriate	20	91	0.338
The font size is appropriate	21	95	0.137
The sections divide appropriately the content	20	92	0.280
The modules divide appropriately the content	22	100	1.000
Content			
The objectives are clearly defined	21	95	0.137
The information is presented in a logical and consistent way	21	95	0.137
There is correlation between images and content	22	100	1.000
There is correlation between videos and content	22	100	1.000
There is correlation between audios and content	22	100	1.000
The proposed homework is relevant	21	95	0.137
The evaluation is relevant	20	91	0.338
Interaction and stimulus			
The RLO generates interest	22	100	1.000
The RLO is easy to use	22	100	1.000
The RLO proposes learning situations	22	100	1.000
The RLO invites to reading	22	100	1.000
The RLO stimulates learning	22	100	1.000
The RLO facilitates the retention of content in memory	21	95	0.137
Recommends using the RLO to other nursing students	22	100	1.000

*Binomial test

## Discussion

This study produced and evaluated the validity of a reusable learning object for teaching the assessment of NANDA Domain 4. Activity/Rest, cardiovascular and pulmonary responses for use in virtual learning environments; we also assessed nursing students' perceptions about the usability of this RLO. Participants, nurses who served as experts and nursing students, positively evaluated the RLO. Although the RLOs offer new opportunities for the teaching and learning process, previous studies in Spanish-speaking countries about the construction and validation of such technologies in the area of nursing were not found, let alone, in the teaching of the assessment of the human responses according to the domains of the NANDA-I Taxonomy.

The construction and validation of educational technologies, such as in the case of the RLOs, require an adequate pedagogical and technical approach; without them, there is a risk of producing technological material free of educational objectives effective.^29^ Hence, the elaboration of the RLO was based on the instructional theory of Robert Gagné, specifically in the Nine Events of instruction.^30^ Gagné's instructional theory seeks to describe the conditions that favor the learning of a particular ability;^31^ it places the student as the focus of the learning process since the student is mainly responsible for the acquisition of knowledge or skills.^31^ In this study, Gagne's events of the instruction provided a sound structure for developing the content of the RLO, which we believe can promote effective learning for the assessment of the cardiovascular and respiratory responses without requiring the constant presence of a teacher.

We highlight that the construction phase of the RLO was the most complex phase of this study; it required the participation of a professional with certified experience in creating RLOs, and another expert in video development. Regarding the content validation phase, it is emphasized that the criterion "aspects addressed by the RLO are important for nursing practice in terms of nursing assessment" obtained the highest score (weighted average 0.97; 100% concordance), see Table 2. In addition, the global Total-VC was satisfactory for the validation process (0.86); however, the specialists made necessary suggestions to improve the RLO.

The content validation of educational technologies allowed us to verify the relevance of the components of the teaching material to the construct they represent; due to the findings in content validation, as in other studies;^25^,^32^ in this study, adjustments to the RLO had to be made before obtaining the final version of it. The content validation demonstrates the importance of this stage in obtaining quality educational resources. On the other hand, the results related to the usability of the RLO from the evaluation carried out by the nursing students were satisfactory, given that all the proportions were greater than 85% (p>0.05). We noted that all students assessed aspects related to interaction and stimulation as appropriate, except for the "Facilitates retention of content in memory" criteria, evidenced by the proportions of 100% concordance.

Current nursing students regularly use the Internet, are digitally fluent, and prefer alternative methodologies to traditional classes; they are a generation that evidence knowledge, skill, and interest in using virtual objects.^33^ Also, as maintained by Windle et al.,^34^ the RLOs can be an effective and popular educational intervention within an aspect of the curriculum that students traditionally find difficult. They are more effective in terms of students' attainment than the traditional lecture format. Such popularity and effectiveness can be explained by la flexibility and accessibility that they provide for study.^34^ In this regard, and as well as exposed by other researchers,^33^ the RLO construed and validated in this study can encourage nursing students to learn autonomously about cardiovascular and pulmonary responses assessment. In the light of the above, we recommend using and making the RLO available to nursing students for assessment of the NANDA Domain 4. Activity/Rest, cardiovascular and pulmonary responses. However, teachers who wish to integrate the RLO in their classrooms must receive preparation based on a fundamental pedagogical approach.^29^

On the other hand, with the advent of the COVID-19 pandemic, advances in technology-mediated teaching became necessary to cover the qualified training needs of nursing students; consequently, the current perspective is the development of new technologies that mediate learning.^35^ In that sense, studies on the development and validation of educational technologies, as is the case in this study, acquire particular relevance because they allow the production of educational resources that make it possible to address content remotely within a friendly learning environment.

It is essential to highlight that one of the strengths of this study was the participation of experts and nursing students who represent the audience for which the RLO was intended. Such participation is crucial because it increases the acceptance and credibility of this educational technology.^36^ Although a rigorous procedure was implemented to develop and validate the proposed RLO, this methodological study has limitations; first, the fragility of the inclusion criteria for experts. In this study, postgraduate training, or publications in the thematic area of the study were not considered, as some authors propose, due to the difficulty of finding nurses in our environment with these characteristics; second, this study having been carried out with nursing students from only one educational institution.

Finally, the authors used only the methodological research approach; therefore, objective measures of improvement in knowledge of assessment of cardiopulmonary responses were not collected; future research should evaluate the effectiveness of the RLO to increase knowledge and improve the performance of nursing students concerning the assessment of the NANDA Domain 4. Activity/Rest, cardiovascular and pulmonary responses.

## Conclusion

The reusable learning object developed in this study is a new resource for teaching the assessment of the NANDA Domain 4. Activity/Rest, cardiovascular and pulmonary responses. It can be considered a valid RLO based on the above results. The validation process included nursing professionals with experience teaching and assessing the health status and nursing students. The suggestions of the nursing specialists and students were considered for the adjustment and final version of the RLO, which can be used for teaching the assessment of cardiovascular and respiratory responses in nursing students.
